# Memory characteristics in mesial temporal lobe epilepsy: Insights from an eye tracking memory game and neuropsychological assessments

**DOI:** 10.1111/cns.14203

**Published:** 2023-04-10

**Authors:** Ling Xiao, Guangpu Zhu, Kailing Huang, Shirui Wen, Li Feng, Beibin Li, Bo Xiao, Ding Liu, Quan Wang

**Affiliations:** ^1^ Department of Neurology, Xiangya Hospital Central South University Changsha 410008 China; ^2^ National Clinical Research Center for Geriatric Disorders, Xiangya Hospital Central South University Changsha Hunan 410008 China; ^3^ Key Laboratory of Spectral Imaging Technology Xi'an Institute of Optics and Precision Mechanics of the Chinese Academy of Sciences Xi'an 710119 China; ^4^ University of Chinese Academy of Sciences Beijing 100049 China; ^5^ Key Laboratory of Biomedical Spectroscopy of Xi'an Xi'an Institute of Optics and Precision Mechanics of the Chinese Academy of Sciences Xi'an 710119 China; ^6^ Paul G. Allen School of Computer Science and Engineering University of Washington Seattle Washington 98195 USA; ^7^ Department of Neurology, the Third Xiangya Hospital Central South University Changsha 410008 China

**Keywords:** eye tracking, hippocampal sclerosis, memory deficit, mesial temporal lobe epilepsy, visual attention

## Abstract

**Aims:**

To compare different patterns of memory impairment in patients with two subtypes of mesial temporal lobe epilepsy (MTLE) and healthy controls.

**Methods:**

Thirty‐five healthy controls and 41 patients with MTLE were recruited, of which 25 patients were diagnosed as hippocampal sclerosis (HS‐MTLE), and the rest 16 patients were lesion‐negative (MRI‐neg MTLE). Participants completed the Wechsler memory assessment and a short‐term memory game on an automated computer‐based memory assessment platform with an eye tracker.

**Results:**

Both the MRI‐neg MTLE and HS‐MTLE groups took longer time to complete the short‐term memory game than healthy controls (*p* < 0.001, Cohen's d = 1.087; *p* = 0.047, Cohen's d = 0.787). During the memory encoding phase, the MRI‐neg MTLE group spent significantly shorter time than healthy controls on the difficult levels with three (*p* = 0.004, Cohen's d = 0.993) and four targets (*p* = 0.016, Cohen's d = 0.858). During the memory decoding phase, the HS‐MTLE group spent less time looking on the targets compared to controls when recalling and finding four targets (*p* = 0.004, Cohen's d = −0.793), while the MRI‐neg MTLE group spent significantly longer time on the distractors and shorter time on the region of interests (ROIs) for all difficulty levels (all *p* < 0.05) than controls. Furthermore, the eye tracking data were correlated with the scores of the Wechsler Memory Scale after Bonferroni correction (*p* < 0.05).

**Conclusion:**

Patients with MRI‐neg MTLE demonstrate impaired memory mostly due to attention deficits, while those with HS‐MTLE show memory impairment with relative sparing of attention. Eye tracking technology has the potential of facilitating the investigation of the mechanism of memory defect in MTLE and can serve as a supplementary neuropsychological tool for clinical diagnosis and long‐term monitoring.

## Graphical abstract

Mesial temporal lobe epilepsy patients without any lesions on MRI (MRI‐neg MTLE) are characterized by impaired memory with significantly attention deficit, while the patients with hippocampal sclerosis (HS‐MTLE) demonstrate isolated memory impairment. Eye tracking technology has the potential of facilitating the investigation of the mechanism of memory defect in MTLE and can serve as a supplementary neuropsychological tool for clinical diagnosis and long‐term monitoring.

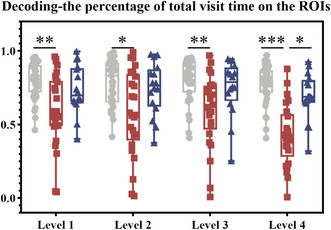

## 1 INTRODUCTION

Mesial temporal lobe epilepsy (MTLE), the most common type of drug‐resistant epilepsy in adults,^1^ usually originates from medial temporal lobe and hippocampal regions.^2^ Patients with MTLE usually present with various cognitive deficits with memory impairment.^3^, ^4^ Although progressive impairment of memory reportedly contributes to difficulties in daily routines in patients with MTLE,^5^ researches focusing on how memory encoding and decoding are affected are lacking.

Functional magnetic resonance imaging (fMRI) studies based on memory tasks have shown that the activation of hippocampus is significantly enhanced during memory encoding activities.^6^, ^7^ The hippocampus holds an essential role in memory formation and consolidation, and it forms a reciprocal signal circuit crucial for translation of temporary hippocampal output to permanent memory.^8^ Hippocampal sclerosis and atrophy are considered responsible for the inability to form new memories, anterograde amnesia, and specific memory deficits.^9^, ^10^

Mesial temporal sclerosis (MTS), also commonly referred to as hippocampal sclerosis (HS), is the most common pathological change of MTLE in adulthood.^11^ Memory impairment in MTLE is thus probably due to structural abnormalities.^12^, ^13^ An alternative view is that patients with MTLE are prone to develop memory impairment regardless of lesion status.^14^, ^15^ A recent study suggests that MTLE patients without any lesions on MRI (MRI‐neg MTLE) are also associated with memory impairment similar to MTLE patients with HS (HS‐MTLE).^15^ An fMRI study conducted by Vaughan et al. found that MRI‐neg MTLE and HS‐MTLE had distinct patterns of network abnormalities.^16^ These results support the notion that memory impairment in MTLE may not always be associated with structural abnormalities but rather an intrinsic deficit of the underlying network malfunction.

To date, the characteristics and underlying mechanism of memory impairment in MTLE remain elusive. Although cognitive scales have been widely applied in memory evaluation in individuals, they still have limitations in early diagnosis and long‐term monitor due to its high degree of subjectivity, low sensitivity, and reproducibility.^17^ In addition, traditional memory scales fail to separate the attention domain from memory formation, while the effect of visual attention on the results of memory tests cannot be ignored, giving rise to mixed and ambiguous conclusions.^18^

Eye tracking technology is featured by its millisecond‐level sampling rate, quantitative measurements, and reliability during the visual monitoring process and has been widely used in cognitive studies.^17^, ^19^ Moreover, this technology can help separate visual attention from memory processing,^20^, ^21^ unfold the pattern of memory encoding and decoding, and elucidate the underlying mechanism of memory deficits in diseases.^22^, ^23^ In the present study, we used the eye tracking‐based automated memory assessment platform,^24^ in combination with Wechsler Memory Scale‐Revised of China (WMS‐RC), aiming to compare memory performance among HS‐MTLE patients, MRI‐neg MTLE patients, and healthy controls. We sought to delineate the patterns of memory impairment in different subtypes of MTLE, and provide a basis to explore underlying mechanisms of memory impairment in MTLE.

## 2 METHODS

### 2.1 Participants

#### 2.1.1 Patients

A total of 41 patients with a diagnosis of MTLE were recruited from the Department of Neurology, Xiangya Hospital. Inclusion criteria were: (1) 18 to 70 years of age, (2) full‐scale IQ ≥70, (3) on regular antiepileptic therapy upon recruitment, and (4) normal or corrected to normal vision and normal hearing. Exclusion criteria: (1) history of neurologic disease other than epilepsy, (2) history of neurosurgery, or (3) subjective memory impairment. All patients underwent clinical assessment including video‐electroencephalography, 3‐tesla MRI, combined attack symptoms, to help identify the epileptogenic foci. Two licensed epileptologists diagnosed and classified the epilepsy types based on International League Against Epilepsy (ILAE) classification of seizures.^25^

Among the patients, 16 (39%) were diagnosed as MTLE with a focal lesion detectable on clinical MRI consistent with HS (HS‐MTLE) (age 31.88 ± 10.47 years), and 25 (61%) without any lesions on MRI (age 31.32 ± 10.66 years). Table 1 presented the demographic and clinical characteristics of the patients in this study.

**TABLE 1:** Demographic and clinical characteristics of participants.

	Controls (*n* = 35)	MRI‐neg MTLE (*n* = 25)	HS‐MTLE (*n* = 16)	*p* Value	Contrasts	*p* Value	Cohen's d
Male, *n* (%)	9 (26)	9 (36)	9 (56)	0.107	—	—	—
Age, y, mean ± SD	32.06 ± 7.82	31.32 ± 10.66	31.88 ± 10.47	0.955	—	—	—
Education, y, mean ± SD	13.77 ± 4.05	11.76 ± 3.31	12.00 ± 3.35	0.085	—	—	—
Age at onset, y, mean ± SD	—	22.98 ± 12.02	20.13 ± 12.46	—	MRI‐neg = HS	0.469	0.233
Duration, y, mean ± SD	—	8.34 ± 6.32	11.75 ± 6.53	—	MRI‐neg = HS	0.104	−0.531
Yearly seizure frequency, mean ± SD	—	43.16 ± 144.60	36.69 ± 44.11	—	MRI‐neg = HS	0.083	0.061
Side of epilepsy foci, *n* (%)				—			
Left	—	8	11				
Right	—	10	4				
Bilateral/Unclear	—	7	1				
No. of antiepileptic drugs, mean ± SD	—	1.24 ± 0.60	1.44 ± 0.51	—	MRI‐neg = HS	0.434	−0.359
Digit Span, mean ± SD	12.13 ± 4.18	9.04 ± 3.40	8.38 ± 3.86	0.001**	MRI‐neg = HS	1.000	0.181
					Controls > MRI‐neg	0.011*	0.811
					Controls > HS	0.007**	0.932
Logical memory, mean ± SD	9.31 ± 2.68	8.72 ± 3.18	6.44 ± 3.08	0.012*	MRI‐neg = HS	0.069	0.728
					Controls = MRI‐neg	1.000	0.201
					Controls > HS	0.010*	0.994
Visual recognition, mean ± SD	10.63 ± 2.65	10.36 ± 1.80	8.81 ± 3.56	0.081	—	—	—

*Note*: Data presented as *n* or mean ± SD. The scores of the Digit Span were calculated by summing the forward and backward subtests and then converting them to equivalence scale scores. The scores of the Logical memory were obtained by converting the sum of scores into equivalent scale scores. The scores of the Visual recognition were summed from the correct recognition of the cards and then converted into equivalent scale scores. Statistical significance between groups is indicated by bold styling and asterisk(s) (*p < 0.05, **p < 0.01, ***p < 0.001).

Abbreviations: HS‐MTLE, mesial temporal lobe epilepsy with hippocampal sclerosis; MRI‐neg MTLE, MRI‐negative mesial temporal lobe epilepsy; y, years; SD, standard deviation; Digit Span, the score of the Wechsler memory scale–Digit Span; Logical memory, the score of the Wechsler memory scale–Logical memory; Visual recognition, the score of the Wechsler memory scale–Visual recognition.

#### 2.1.2 Healthy controls

Thirty‐five healthy controls (age 32.06 ± 7.82 years) matched in age, sex, and level of education to the patients with MTLE were also recruited from the patients' families and the local community. People with neurological or psychiatric diseases, and drug addiction were excluded.

This study received ethical approval from the Ethics Committee of Xiangya Hospital of Central South University, and all participants provided written informed consent to participate in the study.

### 2.2 Wechsler memory assessment

To assess the memory function of the participants, we applied the Digit Span task, the Visual Recognition task and the Logical memory task of the WMS‐RC.^26^, ^27^

Digit Span task: This task measured the verbal working memory and, comprises digit span forward and backward subtests. In the Digit Span Forward subtest, after hearing a sequence of digits dictated by the experimenter, the participant was instructed to repeat the sequence of digits. The test ended until either the maximum length was completed or two consecutive incorrectly repeated sequences of same length occurred. In the Digit Span Backward subtest, the participant was asked to repeat the sequence in reverse order.

Visual Recognition task: This task measured the visual–spatial memory. In this task, eight cards were presented to the participants for 30 s, the participants then were asked to recall the eight presented cards in all 28 cards. These cards include graphics, Chinese characters, and mathematical symbols. The more cards the participants identify, the higher the score.

Logical Memory task: This task measured the verbal episodic memory. The experimenter read two stories to the participant. Then the participant was asked to recall as many details as possible immediately. The more details the participants recalled, the higher the score.

### 2.3 Short‐term memory game

#### 2.3.1 Game design

**FIGURE 1:**
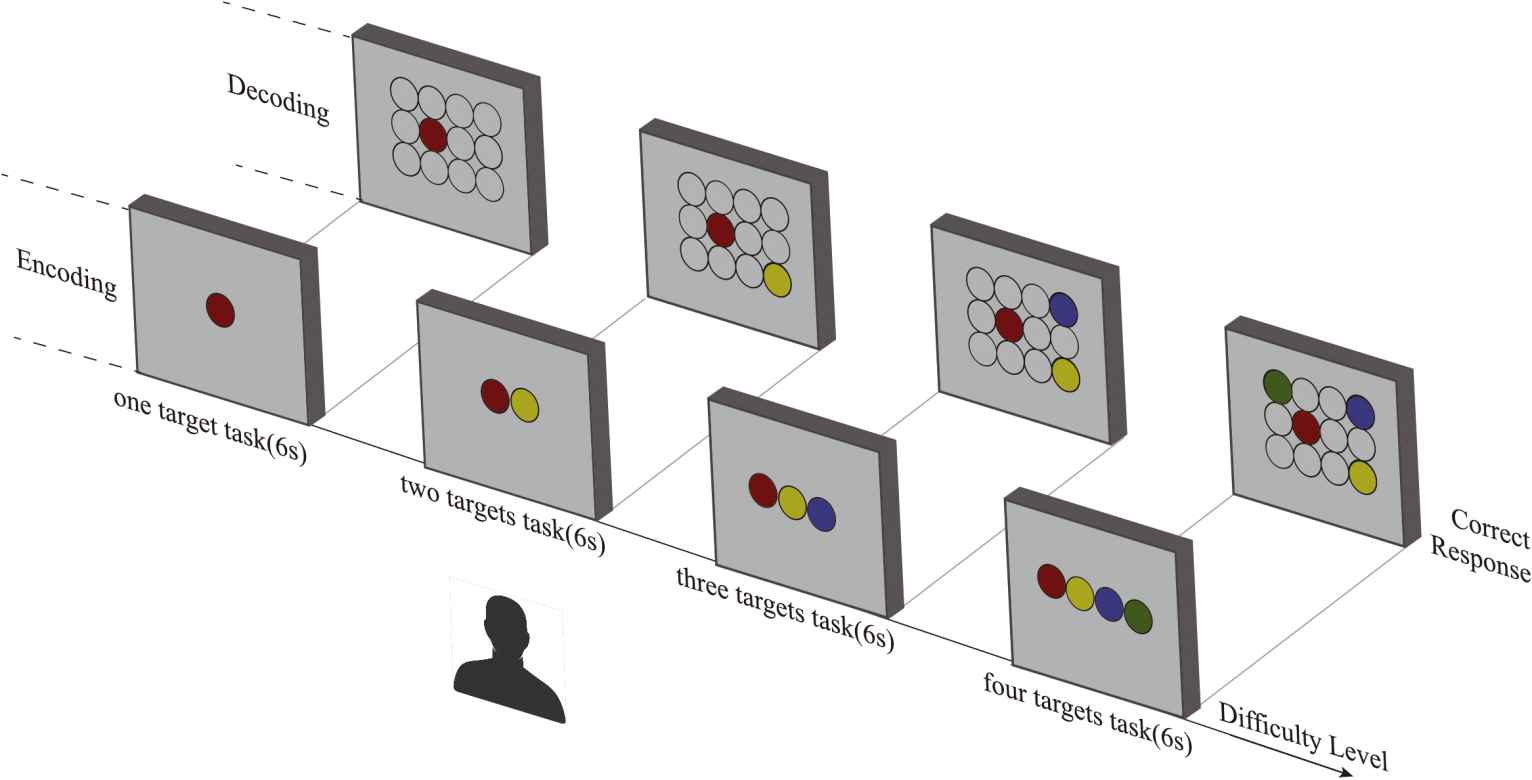
Short‐term memory game with Eye Tracking. After calibration, one target is presented for 6 s on the screen (Encoding), and then, the target disappears and 12 (4 × 3) potential answers instead. The participant needs to recall and click on the target from 4 × 3 objects (Decoding). Memory target(s) from one to four in turn, and the participant must select the target(s) from the previous screen in order to proceed to the next level.

**FIGURE 2:**
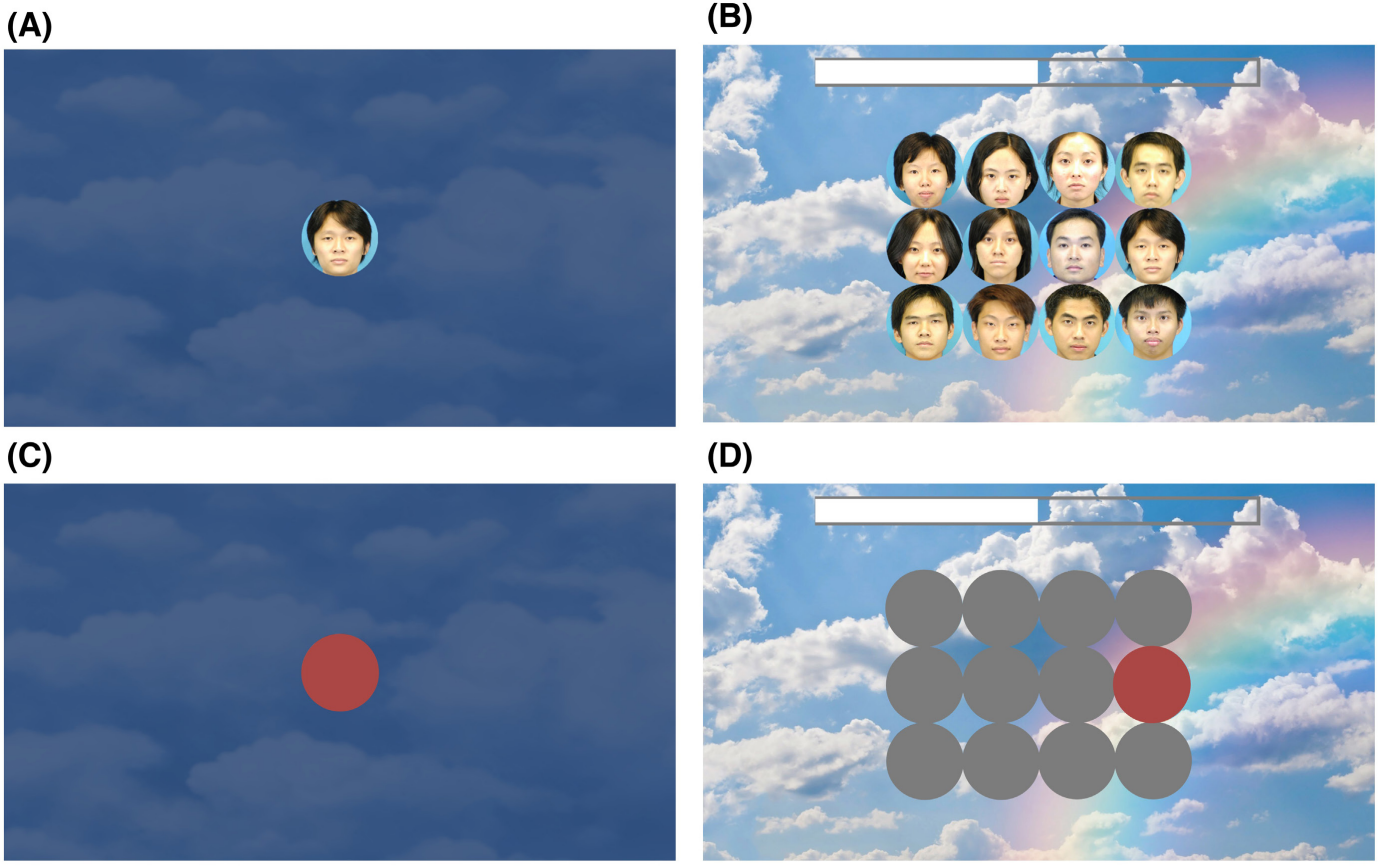
Experiment with stimuli in the short‐term memory game. (A) During the encoding phase, one target is presented to the participants. (B) During the decoding phase, there were 12 potential answers on the screen, and the participant had to click on the target that they had seen during the encoding phase. (C) ROI of the target during the encoding phase. (D) ROIs of the target for recognition (in red) and distractors (in gray).

The automated computer‐based memory assessment platform was adapted from Li et al. as reported in our previous study,^24^ used to measure the short‐term memory. In addition to short‐term memory performance, by applying the Tobii Glass II wearable eye tracker we were realized the real‐time tracking of visual search when performing the short‐term memory game. The stimuli used were 40 human front‐facing images downloaded from the Chinese University of Hong Kong (CUHK) student database (http://mmlab.ie.cuhk.edu.hk/archive/facesketch.html) and 40 fractals downloaded online. The short‐term memory game includes four trials of progressive difficulty, starting with memorizing and recognizing one memory target (level 1) and ending with memorizing and recognizing four memory targets (level 4). A trial began by showing memory targets to the participant for 6 s (the memory encoding phase). In the memory decoding phase, the participant was presented with 12 potential answers (including targets and distractors) arranged in three rows and four columns on the screen (Figures 1, 2). The decoding phase has a time limit of 46 s, after which the game moves on to the next trial. The subjects were asked to acknowledge the memory target(s) by clicking a mouse. They were prompted for a response and after their response was recorded, feedback was provided and they clicked the left mouse button to continue to the next trial. The time taken to complete each level of the memory game and the number of incorrect trials were recorded, and the average completion time was calculated.

#### 2.3.2 Eye tracking

Eye tracking data were collected at a sampling rate of 300 Hz and analyzed with Tobii Pro Lab (version 1.123). The region of interests (ROIs) was defined for memory targets in the memory encoding phase and different images to select in the memory decoding phase. After setting the ROIs, we exported the total visit time on ROIs in the encoding phase, the total visit time on targets, and the total visit time on ROIs in the decoding phase as the output variables. Eye tracking were processed with MATLAB (Version 2015b).

Total visit time on targets (encoding) was defined as the sum of looking duration of all visits in memory target(s) during the memory encoding phase. Total visit time on targets (decoding) was defined as the sum of looking duration of all visits in memory target(s) during the memory decoding phase. Total visit time on distractors (decoding) was defined as the sum of looking duration of all visits in distractors during the memory decoding phase. Total visit time on ROIs (decoding) was the sum of looking duration of all visits in ROIs during the memory decoding phase. And the percentage of total visit time on ROIs was defined as the total visit time on ROIs during the memory decoding phase divided by the game completion time.

### 2.4 Statistical analysis

Statistical analysis was performed using the SPSS software package (ver. 24.0; SPSS Inc., Chicago, Illinois, USA).^28^ The Kolmogorov–Smirnov test was used to evaluate data distribution. Data without a normal distribution or homogeneity of variance would be compared between groups with a nonparametric approach. Characteristics of participants were compared using χ^2^ analyses for categorical variables and one‐way analysis of variance (ANOVA) or Kruskal‐Wallis (K‐W) test for continuous variables among patients with HS‐MTLE, patients with MRI‐neg MTLE, and healthy controls. The scores of the Wechsler memory assessment and the eye tracking data showed a significant departure from a normal distribution and were analyzed with K‐W test. When the K‐W test showed significant difference (*p* < 0.05), Dunn's pairwise test was applied to identify specific group differences. Bonferroni corrected *p* value threshold was employed for post hoc analysis. Spearman's correlation analysis was used to identify correlations between the scores of the Wechsler Memory tasks and the eye tracking data, following Holm–Bonferroni correction. Corrected *p* values of <0.05 were considered significant.

## 3 RESULTS

### 3.1 Demographics

Demographic and clinical characteristics of participants were summarized in Table 1. There were no significant differences in gender, age, or education level among the three groups (*p* > 0.05). The two MTLE groups did not differ in age at epilepsy onset, duration of epilepsy, seizure frequency of epilepsy, or number of antiepileptic drugs (*p* > 0.05).

### 3.2 Wechsler memory assessment

**FIGURE 3:**
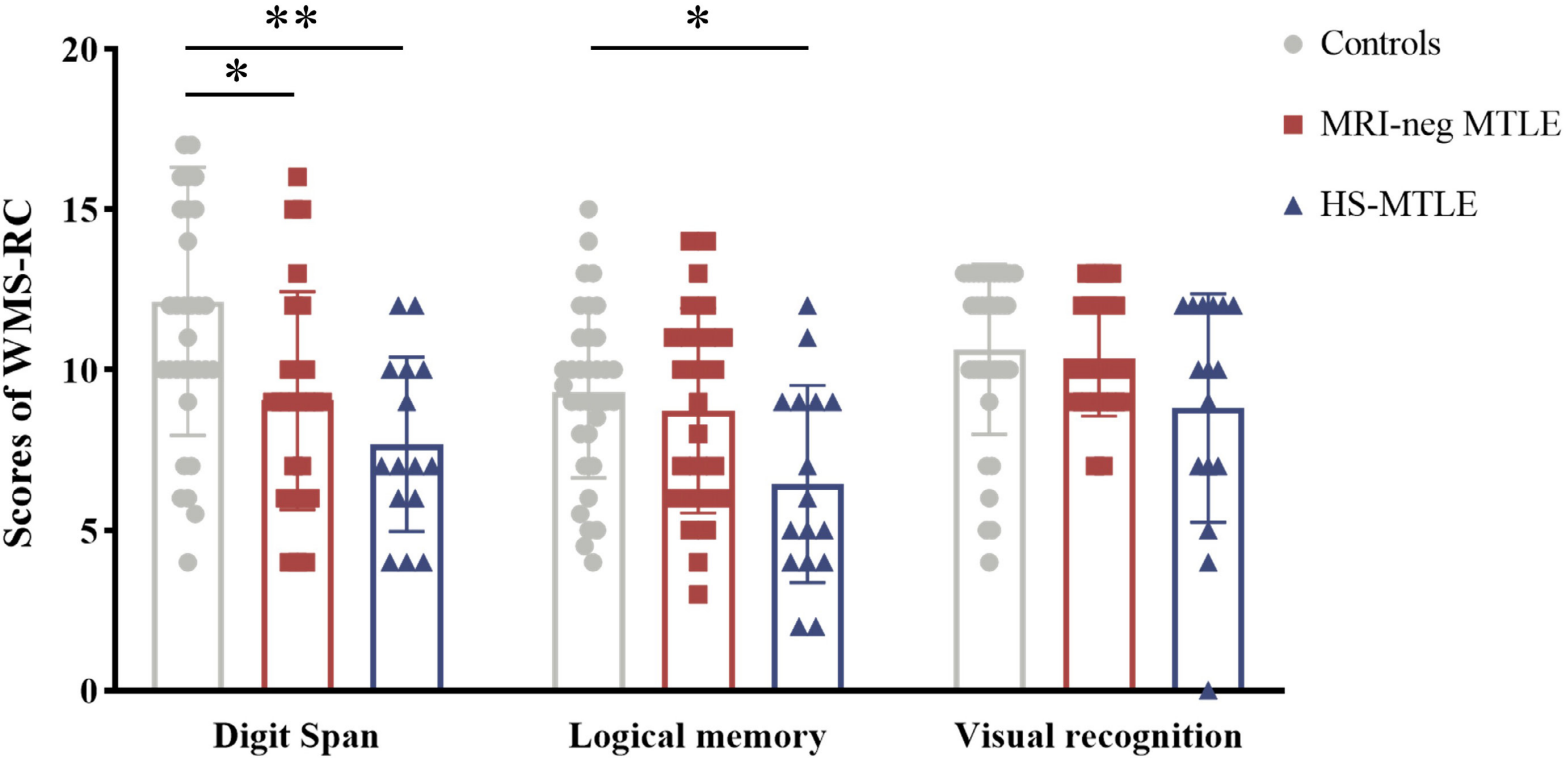
Comparison of scores among the MRI‐neg MTLE group, the HS‐MTLE group and healthy controls: Digit Span task, Logical memory task and Visual Recognition task. Statistical significance for each task between groups is indicated by asterisk(s) (*p < 0.05, **p < 0.01, ***p < 0.001). HS‐MTLE, mesial temporal lobe epilepsy with hippocampal sclerosis; MRI‐neg MTLE, MRI‐negative mesial temporal lobe epilepsy.

In the Digit Span task of WMS‐RC, there were significant differences among the MRI‐neg MTLE group, HS‐MTLE group, and healthy controls (H (2) = 13.030, *p* = 0.001). Both the MRI‐neg MTLE (*p* = 0.011, Cohen's d = −0.811) and HS‐MTLE groups (*p* = 0.007, Cohen's d = −0.932) gave significantly lower scores than healthy controls. The difference of scores in the two MTLE groups was not significant (*p* = 1.000, Cohen's d = 0.181) (Table 1 and Figure 3).

For the Logical Memory task of the WMS‐RC, among the three groups showed significant differences (H (2) = 8.783, *p* = 0.012). The HS‐MTLE group had worse performance than the healthy controls did (*p* = 0.010, Cohen's d = −0.994). The score of the logical memory task in the MRI‐neg MTLE was marginally higher than that in the HS‐MTLE group (*p* = 0.069, Cohen's d = 0.728). There was no difference between the MRI‐neg MTLE and healthy controls in the Logical Memory task (*p* = 1.000, Cohen's d = 0.201) (Table 1 and Figure 3).

We did not find a significant group difference in the performance of the Visual Recognition task among the three groups (H (2) = 5.023, *p* = 0.081) (Table 1 and Figure 3).

### 3.3 Short‐term memory game and eye tracking

**FIGURE 4:**
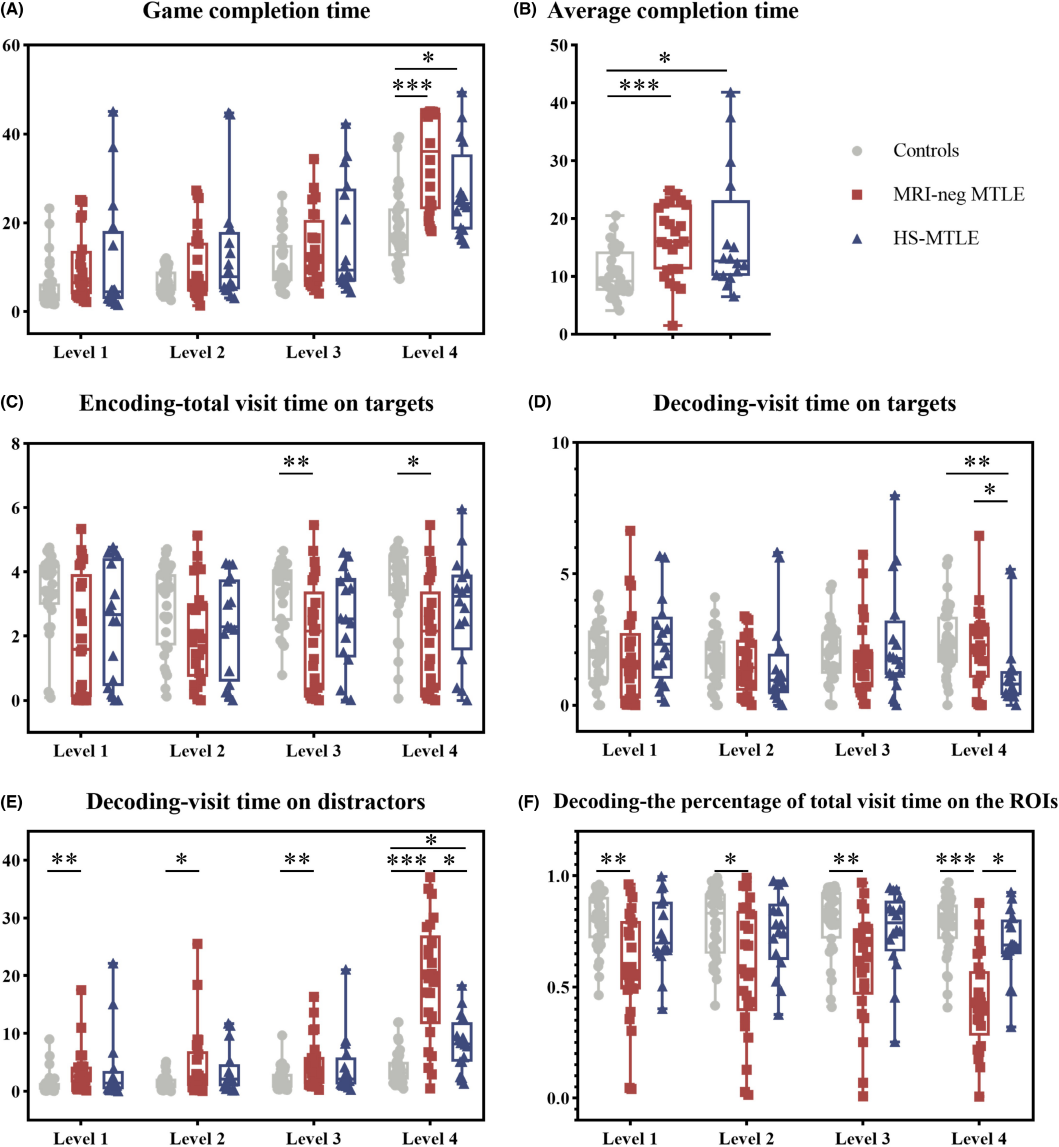
Comparison of (A) Game completion time of the Short‐Term Memory game, (B) Average completion time, (C) Total visit time on targets in the encoding phase, (D) Visit time on targets, (E) Visit time on distractors, and (F) Percentage of total visit time on ROIs in the decoding phase among the MRI‐neg MTLE group, the HS‐MTLE group and healthy controls. Statistical significance for each task between groups is indicated by asterisk(s) (*p < 0.05, **p < 0.01, ***p < 0.001). HS‐MTLE, mesial temporal lobe epilepsy with hippocampal sclerosis; MRI‐neg MTLE, MRI‐negative mesial temporal lobe epilepsy.

Through automated computer‐based assessment with eye tracking, the Kruskal‐Wallis test and post hoc test demonstrated that there were no differences in the number of incorrect trials (Figure S1) and game completion time among the three groups when subjects remembered one, two, or three targets (all *p* > 0.05) (Table 2 and Figure 4A). When four targets had to be memorized, there were significant differences in the completion time of the short‐term memory game among the three groups (H (2) = 21.767, *p* < 0.001). The MRI‐neg MTLE and HS‐MTLE groups both took longer than controls to complete the short‐term memory game (*p* < 0.001, Cohen's d = 1.319; *p* = 0.034, Cohen's d = 0.871), but there was no difference between the two groups (*p* = 0.530, Cohen's d = 0.493) (Table 2 and Figure 4A).

**TABLE 2:** Comparison of eye tracking indicators of participants.

	Controls (*n* = 35)	MRI‐neg MTLE (*n* = 25)	HS‐MTLE (*n* = 16)	*p* Value	Contrasts	*p* Value	Cohen's d
Game completion time in level 1 (s)	5.55 ± 4.99	9.53 ± 7.86	11.11 ± 13.48	0.075			
Game completion time in level 2 (s)	6.83 ± 2.75	9.76 ± 7.88	13.50 ± 13.23	0.278			
Game completion time in level 3 (s)	10.88 ± 5.85	13.43 ± 8.52	16.37 ± 12.61	0.521			
Game completion time in level 4 (s)	19.03 ± 8.37	32.69 ± 12.64	26.87 ± 10.30	<0.001***	MRI‐neg = HS	0.530	0.493
					Controls < MRI‐neg	<0.001***	−1.319
					Controls <HS	0.034*	−0.871
Average completion time (s)	10.57 ± 4.10	16.35 ± 6.30	16.96 ± 10.73	<0.001***	MRI‐neg = HS	1.000	−0.069
					Controls < MRI‐neg	<0.001***	−1.087
					Controls <HS	0.047*	−0.787
Encoding							
Total visit time on target in level 1 (s)	3.35 ± 1.24	1.93 ± 1.97	2.46 ± 1.88	0.053	—	—	—
Total visit time on targets in level 2 (s)	2.95 ± 1.32	2.07 ± 1.56	2.31 ± 1.60	0.101	—	—	—
Total visit time on targets in level 3 (s)	3.37 ± 1.01	2.00 ± 1.67	2.52 ± 1.60	0.005**	MRI‐neg = HS	1.000	−0.318
					Controls > MRI‐neg	0.004**	0.993
					Controls = HS	0.215	0.635
Total visit time on targets in level 4 (s)	3.58 ± 1.25	2.31 ± 1.68	2.87 ± 1.75	0.017*	MRI‐neg = HS	1.000	−0.326
					Controls > MRI‐neg	0.016*	0.858
					Controls = HS	0.350	0.467
Decoding							
Visit time on targets in level 1 (s)	2.20 ± 1.62	1.78 ± 1.73	2.47 ± 1.66	0.248	—	—	—
Visit time on targets in level 2 (s)	1.76 ± 0.99	1.52 ± 1.08	1.54 ± 1.77	0.231	—	—	—
Visit time on targets in level 3 (s)	1.96 ± 1.11	1.77 ± 1.42	2.41 ± 2.15	0.455	—	—	—
Visit time on targets in level 4 (s)	2.41 ± 1.26	2.23 ± 1.48	1.29 ± 1.55	0.005**	MRI‐neg > HS	0.023*	0.620
					Controls = MRI‐neg	1.000	0.131
					Controls > HS	0.004**	0.793
Visit time on distractors in level 1 (s)	1.28 ± 1.84	3.66 ± 3.80	3.73 ± 6.18	0.001**	MRI‐neg = HS	0.295	−0.014
					Controls < MRI‐neg	0.001**	−0.844
					Controls = HS	0.419	−0.654
Visit time on distractors in level 2 (s)	1.43 ± 1.32	4.60 ± 6.02	3.46 ± 3.93	0.019*	MRI‐neg = HS	1.000	0.215
					Controls < MRI‐neg	0.023*	−0.792
					Controls = HS	0.212	−0.833
Visit time on distractors in level 3 (s)	2.17 ± 1.95	5.93 ± 7.19	4.17 ± 5.21	0.009**	MRI‐neg = HS	0.817	0.271
					Controls < MRI‐neg	0.007**	−0.774
					Controls = HS	0.430	−0.604
Visit time on distractors in level 4 (s)	4.05 ± 2.93	19.21 ± 10.25	8.35 ± 4.77	<0.001***	MRI‐neg > HS	0.040*	1.268
					Controls < MRI‐neg	<0.001***	−2.178
					Controls < HS	0.039*	−1.196
The percentage of total visit time on the ROIs in level 1	0.80 ± 0.13	0.59 ± 0.25	0.74 ± 0.17	0.003**	MRI‐neg = HS	0.347	−0.702
					Controls > MRI‐neg	0.002**	1.054
					Controls = HS	0.625	0.396
The percentage of total visit time on the ROIs in level 2	0.79 ± 0.16	0.58 ± 0.29	0.74 ± 0.18	0.013*	MRI‐neg = HS	0.275	−0.663
					Controls > MRI‐neg	0.011*	0.897
					Controls = HS	1.000	0.294
The percentage of total visit time on the ROIs in level 3	0.80 ± 0.15	0.60 ± 0.24	0.75 ± 0.20	0.001**	MRI‐neg = HS	0.107	−0.679
					Controls > MRI‐neg	0.001**	0.999
					Controls = HS	1.000	0.283
The percentage of total visit time on the ROIs in level 4	0.78 ± 0.14	0.43 ± 0.22	0.69 ± 0.17	<0.001***	MRI‐neg < HS	0.012*	−1.323
					Controls > MRI‐neg	<0.001***	1.898
					Controls = HS	0.365	0.578

*Note*: Data presented as mean ± SD. Statistical significance between groups is indicated by bold styling and asterisk(s) (*p < 0.05, **p < 0.01, ***p < 0.001).

Abbreviations: HS, mesial temporal lobe epilepsy with hippocampal sclerosis; MRI‐neg, MRI‐negative mesial temporal lobe epilepsy; SD, standard deviation; s, seconds.

The average completion time differed significantly among the three groups (H (2) = 15.618, *p* < 0.001). Similarly, both the MRI‐neg MTLE and HS‐MTLE groups took longer to complete the memory game than healthy controls (*p* < 0.001, Cohen's d = 1.087; *p* = 0.047, Cohen's d = 0.787). And there was no difference in average completion time between the MRI‐neg MTLE and HS‐MTLE groups (*p* = 1.000, Cohen's d = 0.069) (Table 2 and Figure 4B).

During the memory encoding phase, the total visit time on targets was not different among the three groups when focusing on one or two targets (all *p* > 0.05), but showed a significant difference when focusing on three and four targets (H (2) = 10.773, *p* = 0.005; H (2) = 8.113, *p* = 0.017). Compared with the healthy control group, the MRI‐neg MTLE group spent significantly shorter time on the three targets (*p* = 0.004, Cohen's d = −0.993) and four targets (*p* = 0.016, Cohen's d = −0.858), and the differences between the HS‐MTLE group with the MRI‐neg MTLE group or with the healthy controls were not significant (all *p* > 0.05) (Table 2 and Figure 4C).

During the memory decoding phase, there was no difference for the total visit time on targets when recalling and finding one, two, or three targets (all *p* > 0.05). However, the difference of the time recalling and finding four targets reached a statistical significance (H (2) = 10.804, *p* = 0.005). The HS‐MTLE group spent less time on the targets than both the healthy control (*p* = 0.004, Cohen's d = −0.793) and the MRI‐neg MTLE groups did (*p* = 0.023, Cohen's d = 0.620). There was no difference between MRI‐neg MTLE group and healthy control group (*p* = 1.000, Cohen's d = 0.131) (Table 2 and Figure 4D).

During decoding, eye tracking data revealed that the MRI‐neg MTLE group spent more time on distractors than controls at all four difficulty levels (all *p* < 0.05, Table 2). There were no differences in visit time on distractors between the HS‐MTLE group and the MRI‐neg MTLE group or with the healthy controls during the process of trying to find one, two, or three targets (all *p* > 0.05), but the difference when trying to find four targets is significant (*p* = 0.040, Cohen's d = −1.268; *p* = 0.039, Cohen's d = 1.196) (Table 2 and Figure 4E).

The percentage of total visit time on the ROIs was significant different among the three groups for all four difficulty levels (all *p* < 0.05, Table 2). The percentage of total visit time on the ROIs of the MRI‐neg MTLE group was significantly less than that the healthy control group for all difficulty levels (all *p* < 0.05, Table 2). There were no differences between HS‐MTLE group and MRI‐neg MTLE group in the percentage of total visit time on the ROIs when recalling one, two, or three targets (all *p* > 0.05), but the difference when recalling and finding four targets is significant (*p* = 0.012, Cohen's d = +1.323) (Table 2 and Figure 4F). The differences between the HS‐MTLE group with the healthy controls were not significant in the percentage of total visit time on the ROIs for all four difficulty levels (Table 2 and Figure 4F).

### 3.4 Correlation of WMS‐RC and eye tracking

**FIGURE 5:**
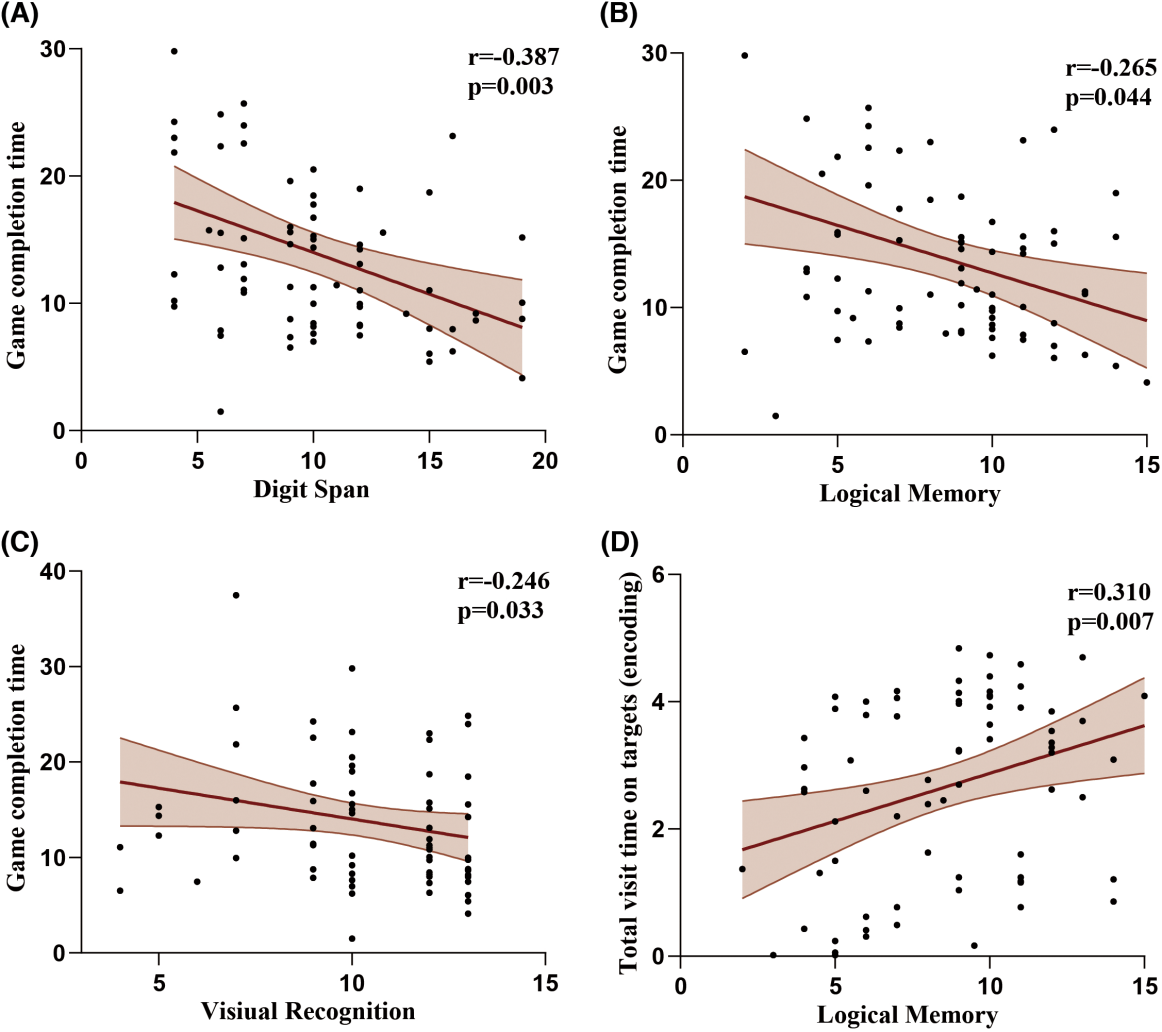
Correlations between game completion time and the scores of the (A) Wechsler Digit Span task, (B) Logical memory task and (C) Visual recognition task. Correlations between Total visit time on targets in the encoding phase and the scores of Digit Span task (D). Red lines indicate linear fits. Light red regions indicate standard error of the means.

We analyzed the correlation between the scores of the WMS‐RC and the eye tracking performance of all participants. Results of Spearman correlation showed that the scores of Digit Span task (rs(75) = −0.387, *p* = 0.003), Logical memory task (rs(75) = −0.265, *p* = 0.044), and Visual recognition task (rs(75) = −0.246, *p* = 0.033) of the WMS‐RC were negatively correlated with the memory game completion time (Figure 5). The performance of the participants in the Logical Memory task was positively correlated with the total visit time on targets in the memory encoding phase (rs(74) = 0.310, *p* = 0.007) (Figure 5). However, there was no correlation between the other WMS‐RC scales scores and eye tracking data (all *p* > 0.05).

## 4 DISCUSSION

This is the first study to investigate characteristics of memory impairment in MTLE subtypes using eye tracking technology. We confirmed that MTLE patients exhibited memory impairment based on the lower scores of WMS‐RC and the longer completed time of the short‐term memory game. By further comparing the eye tracking data, we found that MRI‐neg MTLE patients had shorter total visit time on targets during the encoding phase, longer visit time on distractors and lower percentage of total visit time on the ROIs during the decoding phase, while there was no difference in these two statistics between the HS‐MTLE group and the healthy group. Meanwhile, HS‐MTLE patients spent less visit time on the targets in the memory decoding phase. We, therefore, propose that patients with MRI‐neg MTLE and patients with HS‐MTLE have different mechanisms and patterns of memory deficits. While patients with MRI‐neg MTLE have memory impairment with significantly attention deficits, the patients with HS‐MTLE tend to show memory impairment with relative sparing of attention.

Our results demonstrated that HS‐MTLE patients showed memory deficits, especially during execution of the more complexed memory tasks. In the Digit Span and Logical Memory task of WMS‐RC, the worse performance of HS‐MTLE group denotes memory impairment and lack of complicated analytical logic. However, the HS‐MTLE group performed similar to the healthy controls in the Visual Recognition task, probably due to the relatively simple visual stimulus to remember. In line with the results of the Digit Span and Logical Memory task in the WMS‐RC and previous visual short‐term memory studies,^29^, ^30^ HS‐MTLE patients took longer time to complete our short‐term memory game than controls, which again confirmed the memory impairment. More specificity, during our short‐term memory game, HS‐MTLE patients and controls performed similarly when there were fewer targets to memorize, the differences turned up only when the participants were required to recall as many as four targets. When the level of task difficulty increased with the number of targets, the HS‐MTLE patients displayed remarkable impairment of memory formation, especially in the decoding phase. We propose that the memory impairment in these patients may be too mild or too early to be identified by simple tasks.

Currently, whether MRI‐neg MTLE patients have memory deficit remains controversial.^13^, ^15^, ^31^ Our results suggest that the memory impairment in MRI‐neg MTLE patients may be related to their attentional deficit. On one side, MRI‐neg MTLE patients had lower scores in the Digit Span task of WMS‐RC and took longer to complete our short‐term memory game than controls, which confirmed memory defect in MTLE patients without imaging abnormalities. We also found that MRI‐neg MTLE patients spent less time scanning the ROIs and more time on distractors. It indicated they may not concentrate on the memory targets during either the memory encoding or decoding phases, inferring impaired sustained attention functions that cause worse memory test performances. Epilepsy is considered a network disease.^32^, ^33^ Even without lesions, the epileptic discharge of MRI‐neg MTLE can originate from temporal lobe and hippocampus and lead to memory impairment.^18^ The discharges may also generalize to the frontal lobe through major projection fiber tracts of temporal stem,^34^ causing network involvement in the frontal lobe, especially the attention network, resulting in a combined loss of attention and memory.^35^

With high task difficulty of three or four targets to remember, the two subtypes of MTLE displayed different characteristics of memory deficits. Note that although both MTLE groups exhibited lower scores in memory tasks of WMS‐RC scale results and our game completion time, they displayed different trends when separating the short‐term memory game into encoding and decoding stages. With high task difficulty of four targets, the patients with MRI‐neg MTLE had longer visit time on distractors and decreased percentage of time scanning the ROIs, which may imply their insufficient attention, while the patients with HS‐MTLE had decreased visit time on targets that suggest recognition memory deficits. However, there is no difference in eye tracking results for the three targets or less between HS‐MTLE and MRI‐neg MTLE groups, suggesting that suitable difficult level and sensitive tasks are necessary for the early recognition of memory impairment.

We propose the following possible mechanism for the different characteristics of memory deficits in the two MTLE groups. Attention usually interfered with the performance of memory tasks. Attention requires normal neocortical activation via direct and indirect ascending excitatory projects from subcortical regions.^36^ From a developmental perspective, seizure activity and specific pathological features begin in the hippocampus, and may initially remain confined to the hippocampus and does not interfere with normal cortical activity in the early state,^37^ resulting in predominant memory impairment with the attention domain spared. Notably, we find that the MRI‐neg MTLE patients show memory impairment with particularly significant attention deficits. Several observational studies have compared FDG‐PET findings between patients with MRI‐neg MTLE and HS‐MTLE, interrogating at sublobar patterns of PET hypometabolism using semiquantitative analyses.^38^, ^39^ They have identified that in MRI‐neg MTLE, the hypometabolism area frequently extends beyond the mesial and anterior temporal structures to affect the posterior temporal and even extratemporal regions. This finding may suggest a more extensive epileptogenic network in MRI‐neg MTLE compared to HS‐MTLE.^40^ These evidences have supported the notion that MRI‐neg MTLE is not just an early HS‐MTLE, but appears to be a heterogeneous group encompassing temporal and extratemporal lobes, with more profound network involvement especially in frontal and parietal lobes, resulting attention or other related cognitive impairment.

There is also a strong correlation between the scores of the Logical Memory task and the total visit time on targets of eye tracking in the memory encoding phase. These results are consistent with previous studies that found that eye tracking findings correlate well with traditional cognitive assessment scales.^41^ Memory games based on eye tracking can be an alternative option of neuropsychological assessment that is able to distinguish between the encoding and the decoding phases, differentiate attention from memory deficit, and delineate different memory defect patterns.

Eye tracking technology has been increasingly utilized in cognitive assessment of patients with neurodegenerative diseases such as Alzheimer's disease,^42^, ^43^ but its application for detecting cognitive impairment of epilepsy patients is still rare. We have found the correlation between the WMS‐RC test scores and the game completion time and the correlation between the eye tracking data and the test scores, which showed that eye tracking‐based cognitive tests are potential to be an adjunct for the cognitive assessment in the patients with MTLE.^17^, ^44^ Moreover, eye tracking technology has considerably higher sensitivity than assessment scales^17^ by separating visual attention from memory process and extract differential patterns of memory impairment between MTLE subgroups. Our previous studies have confirmed that the eye tracking‐based automated memory assessment platform can be an assistive tool to evaluate visual attention and memory performance in temporal lobe epilepsy. Eye tracking technology can help separate attention factor from memory processing, motivated by which we have identified different patterns of memory deficits in the MTLE subtypes in this study. We propose that eye tracking‐based cognitive tests are a promising supplementary tool for neuropsychological evaluation, especially in the early diagnosis of and quantitative assessment for the cognitive function.

## 5 LIMITATIONS

Our present work is limited in several ways. Firstly, the results of this study are limited to the sample size of the study. Prospective studies with larger sample sizes are required to further our understanding of the complex mechanism for memory dysfunction in MTLE patients. Secondly, patients with intellectual disability are excluded from the present study. This is an important subgroup of patients with epilepsy, without whom the universality of neuropsychological research results would be questioned. Moreover, the lateralization of MTLE is an important factor in that left MTLE is probably impaired on verbal memory and right MTLE on nonverbal memory impairment. Future studies should aim to explore different characteristics of memory function in patients with different side lesions. Finally, the influence of antiepileptic drugs on cognition is unfortunately an inevitable confound limiting the statistical power in our study, newly diagnosed patients should be enrolled to exclude the interfering effects of drugs.

## 6 CONCLUSION

In conclusion, by the eye tracking‐based memory tasks and traditional assessment scales, we find that patients with MRI‐neg MTLE are characterized by impaired memory with significantly attention deficit, while the patients with HS‐MTLE demonstrate isolated memory impairment. Our results also suggest that eye tracking technology can be a supplementary clinical tool to provide insight into cognitive processes, and holds promise for early diagnosis and intervention in cognitive impairment associated with neurological diseases.

## CONFLICT OF INTEREST STATEMENT

The authors declare no conflict of interest.

## Supporting information


Figure S1.
Table S1.

## Data Availability

Data are available to researchers on request for purposes of reproducing the results or replicating the procedure by directly contacting the corresponding authors.
