# Supplementary material for: Memory characteristics in mesial temporal lobe epilepsy: Insights from an eye tracking memory game and neuropsychological assessments
Source: CNS Neurosci Ther. 2023 Apr 10;29(9):2621–33. doi: 10.1111/cns.14203 (PMC10401175; doi:10.1111/cns.14203)

**Memory characteristics in mesial temporal lobe epilepsy: Insights from an eye tracking memory game and neuropsychological assessments**

**Supplemental Information**

**Reference results**

**Short-term memory game** Compared to controls, patients had a higher number of incorrect trials, but the differences were not statistical significance (H (2) = 3.552, p = 0.169).

**Supplemental table** Comparison of the number of incorrect trials of participants.

|  | Controls(n=35) | MRI-neg MTLE(n=25) | HS-MTLE (n=16) | P value |
| --- | --- | --- | --- | --- |
| Number of incorrect trials | 7.86±4.79 | 9.84±6.01 | 12.75±8.65 | 0.169 |

**Supplemental figure** Comparison of the number of incorrect trials among the MRI-neg MTLE group, the HS-MTLE group and healthy controls.


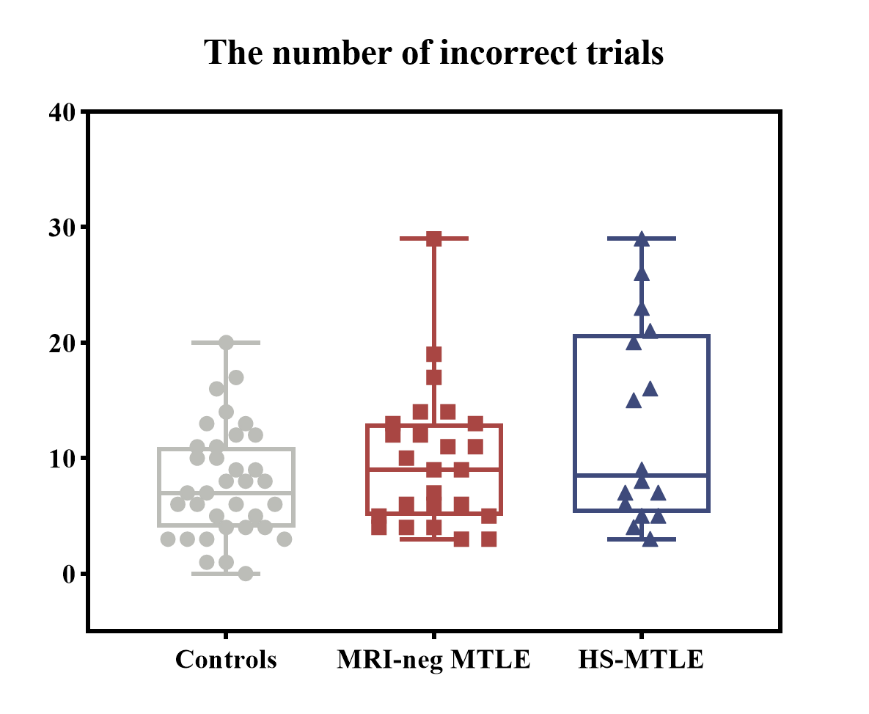

Supplement: Supplementary file 1 — Figure S1. Table S1. [file CNS-29-2621-s001.docx]
